# Correction: SEC24A deficiency lowers plasma cholesterol through reduced PCSK9 secretion

**DOI:** 10.7554/eLife.112375

**Published:** 2026-06-24

**Authors:** Xiao-Wei Chen, He Wang, Kanika Bajaj, Pengcheng Zhang, Danjun Ma, Yongsheng Bai, Hui-Hui Liu, Elizabeth Adams, Andrea Baines, Genggeng Yu, Maureen A Sartor, Bin Zhang, Zhengping Yi, Jiandie Lin, Stephen G Young, Randy Schekman, David Ginsburg

**Keywords:** Mouse

Chen X-W, Wang H, Bajaj K, Zhang P, Meng Z-X, Ma D, Bai Y, Liu H-H, Adams E, Baines A, Yu G, Sartor MA, Zhang B, Yi Z, Lin J, Young SG, Schekman R, Ginsburg D. 2013. SEC24A deficiency lowers plasma cholesterol through reduced PCSK9 secretion. *eLife* **2**:e00444. doi: 10.7554/eLife.00444.

Published 9 April 2013

We have been made aware through a notification from PubPeer that, in the above publication, the lung image in Figure 1 (the lower right section of panel F) appears to be a duplicate of an image in an earlier publication from our research group (lower right panel from Figure 5 in *Thrombosis Research*, 123:785–792, 2009, PMID: 18774162). The lung histologies in both animals (a wild-type control mouse in the 2009 paper and a *Sec24a^gt/gt^* mouse in the 2013 *eLife* paper) were entirely normal, as determined by the expert pathologist who extensively reviewed the tissues in both animals, prepared both figures, and is a co-author on both papers. We have concluded that the error likely occurred during preparation of the *eLife* figure, at which time the earlier image of normal lung histology was accidentally inserted in place of an image of similar normal lung histology from the *Sec24a^gt/gt^* mouse. We sincerely apologize for this error.

To correct this error in the *eLife* manuscript, panel F from Figure 1 has been removed, and the description of panel F in the Figure 1 legend has also been removed. Also, the text in the second paragraph of ***Results*** has been modified as follows:

Corrected text:

Gross and routine microscopic survey of multiple tissues failed to identify any obvious morphologic abnormalities in adult *Sec24a^gt/gt^* mice.

Original text:

Gross and routine microscopic survey of multiple tissues failed to identify any obvious morphologic abnormalities in adult *Sec24a^gt/gt^* mice (Figure 1F).

The corrected Figure 1 (with Panel F removed) is shown here:

**Figure 1:**
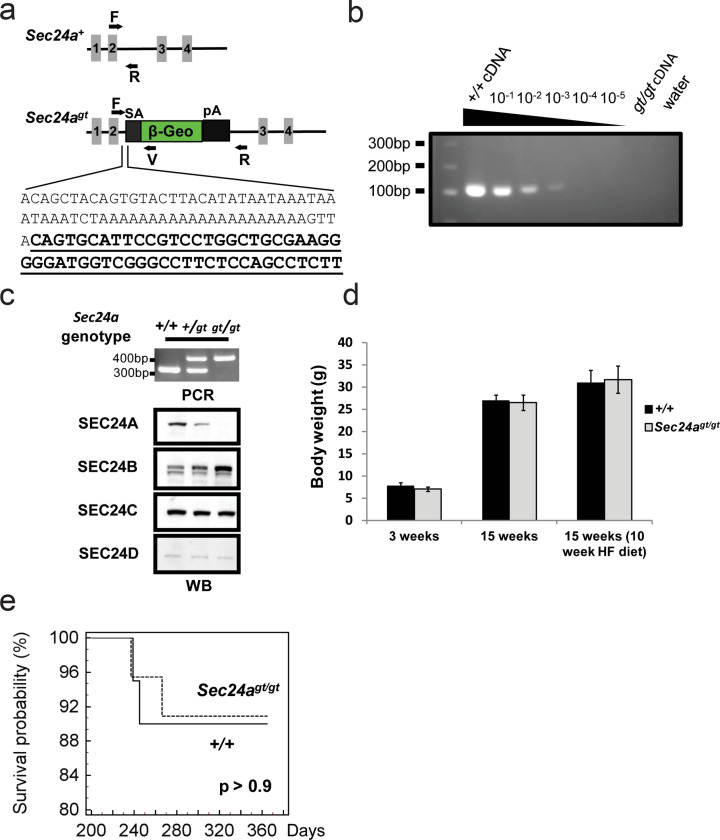


Corrected Figure 1 legend text:

Figure 1. SEC24A null mice are viable and exhibit normal survival and development. (A) Schematic of the first *Sec24a* mutant allele (*Sec24agt*). Gray blocks represent exons with specific numbers indicated. SA, splice acceptor cassette; *β*-Geo, *β*-galactosidase-*neo* fusion; pA, poly-adenylation sequence. F, R, and V represent genotyping primers. Bottom, sequence of *Sec24agt* gene trap insertion junction; sequence of the gene trap cassette is underlined. (B) RT-PCR detection of splicing between exons 2 and 3 in *Sec24agt/gt* mice. Liver cDNA of wild type mice was serially diluted into liver cDNA of *Sec24agt/gt* mice as indicated and used as template for PCR with primers *Sec24a*-Exon2 and *Sec24a*-Exon3 (see primer sequences). (C) Loss of SEC24A protein in *Sec24agt/gt* mice. Upper panel, PCR genotyping; lower panel, immunoblotting of brain protein extracts from mice with the genotypes indicated at the top, using the indicated SEC24A-D antibodies. (D) Body weights of SEC24A-deficient and wild type control mice. HF, high fat diet. Error bars represent SEM (standard error of the mean). At least six mice were included in each group at each time point. (E) Kaplan Meier plot for survival of SEC24A-deficient mice (n=20) and littermate controls (n=15).

The originally published Figure 1 is shown for reference:

**Figure 2:**
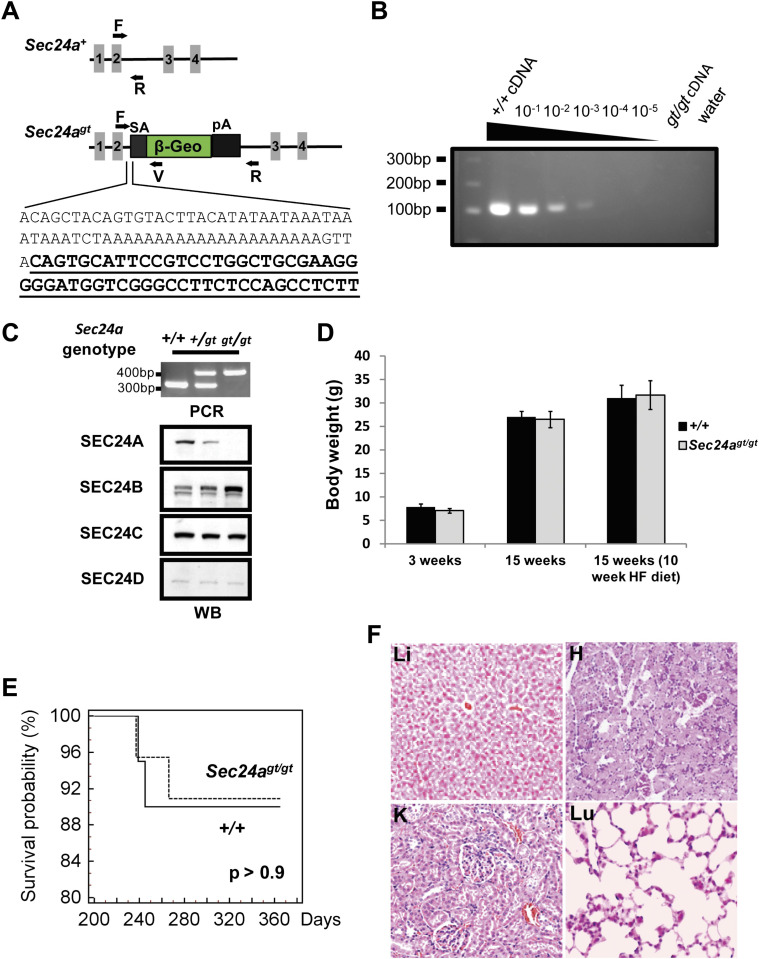


Original Figure 1 legend text:

Figure 1. SEC24A null mice are viable and exhibit normal survival and development. (A) Schematic of the first *Sec24a* mutant allele (*Sec24agt*). Gray blocks represent exons with specific numbers indicated. SA, splice acceptor cassette; *β*-Geo, *β*-galactosidase-*neo* fusion; pA, poly-adenylation sequence. F, R, and V represent genotyping primers. Bottom, sequence of *Sec24agt* gene trap insertion junction; sequence of the gene trap cassette is underlined. (B) RT-PCR detection of splicing between exons 2 and 3 in *Sec24agt/gt* mice. Liver cDNA of wild type mice was serially diluted into liver cDNA of *Sec24agt/gt* mice as indicated and used as template for PCR with primers *Sec24a*-Exon2 and *Sec24a*-Exon3 (see primer sequences). (C) Loss of SEC24A protein in *Sec24agt/gt* mice. Upper panel, PCR genotyping; lower panel, immunoblotting of brain protein extracts from mice with the genotypes indicated at the top, using the indicated SEC24A-D antibodies. (D) Body weights of SEC24A-deficient and wild type control mice. HF, high fat diet. Error bars represent SEM (standard error of the mean). At least six mice were included in each group at each time point. (E) Kaplan Meier plot for survival of SEC24A-deficient mice (n=20) and littermate controls (n=15). (F) Histology of several tissues from *Sec24agt/gt* mice. Li, liver; H, heart; K, kidney; Lu, lung.

The article has been corrected accordingly.

